# Safety and efficacy of pegylated recombinant human granulocyte colony-stimulating factor during concurrent chemoradiotherapy for small-cell lung cancer: a retrospective, cohort-controlled trial

**DOI:** 10.1186/s12885-022-09644-8

**Published:** 2022-05-13

**Authors:** Cunliang Wang, Shouhui Zhu, Chuanwang Miao, Yu Wang, Jiazhen Chen, Shuanghu Yuan, Xudong Hu

**Affiliations:** 1 Shandong First Medical University, Jinan, 250000 Shandong China; 2 Department of Radiation Oncology, Shandong Cancer Hospital, Shandong Cancer Hospital affiliated to Shandong First Medical University, No. 440 Jiyan Road, Jinan, 250117 Shandong China; 3 Department of Radiation Oncology, Shandong Second Provincial General Hospital, Jinan, 250022 Shandong China

**Keywords:** Febrile neutropenia, Hematological toxicity, PEG-rhG-CSF, Radiotherapy, Survival time

## Abstract

**Objective:**

To investigate pegylated recombinant human granulocyte colony-stimulating factor (PEG-rhG-CSF) safety and efficacy in preventing hematological toxicity during concurrent chemoradiotherapy (CCRT) for small-cell lung cancer (SCLC).

**Methods:**

We retrospectively assessed 80 SCLC patients treated with CCRT from January 2013 to December 2018 who received PEG-rhG-CSF within 48 hours after the end of chemotherapy, defined as prophylactic use, as the experimental group. An additional 80 patients who were not treated with PEG-rhG-CSF were matched 1:1 by the propensity score matching method and served as the control group. The main observations were differences in hematological toxicity, neutrophil changes, febrile neutropenia (FN) incidence and adverse reactions. Progression-free survival (PFS) and overall survival (OS) were analyzed with regular assessment and follow-up.

**Results:**

The leukocyte, neutrophil, erythrocyte, and platelet counts and hemoglobin level decreased after CCRT, but the experimental group had slightly higher leukocyte and neutrophil counts than the control group (*P* < 0.05). The incidences of grade III-IV leukopenia (18.75% vs. 61.25%) and neutropenia (23.75% vs. 67.5%) in the experimental group were significantly lower than those in the control group (*P* < 0.05). The absolute neutrophil count was 4.17 ± 0.79 (× 10^9^/L) on day 1 and peaked 6.81 ± 2.37 (× 10^9^/L) on day 10 in the experimental group; the value in the control group was 2.81 ± 0.86 (× 10^9^/L) on day 1. It decreased significantly and reached the minimum 0.91 ± 0.53 (× 10^9^/L) on day 10 (*P* < 0.05). The experimental group had a lower FN incidence than the control group (*P* < 0.05). There was also no significant acute esophagitis or pulmonary toxicity. The treatment had no significant effect on PFS (11.4 months vs. 8.7 months, *P* = 0.958) or OS (23.9 months vs. 17.3 months, *P* = 0.325) over an 18.6-month median follow-up time.

**Conclusion:**

PEG-rhG-CSF has good efficacy and safety in preventing hematological toxicity in SCLC patients during CCRT and has no significant effects on PFS or OS.

**Supplementary Information:**

The online version contains supplementary material available at 10.1186/s12885-022-09644-8.

## Introduction

Small-cell lung cancer (SCLC) accounts for approximately 15% of all lung cancer cases. It is a poorly differentiated and highly malignant tumor with partial neuroendocrine characteristics, rapid growth, strong invasiveness, early metastasis and poor prognosis [1]. Limited-stage SCLC accounts for approximately 30% of all SCLC cases at the time of diagnosis [2]. Concurrent chemoradiotherapy (CCRT) can reduce tumor volume, suppress tumor blood supply and provide a synergistic sensitizing effect, and it is used to treat a wide range of solid tumors. CCRT is the standard treatment for patients with SCLC [3]. It can improve the efficacy of the treatment and produces a significant survival benefit. However, it can also increase the incidence of acute blood toxicity, which can reduce the dose of chemotherapy and delay treatment. Severe neutropenia can easily induce febrile neutropenia (FN), infectious toxic shock and death, which directly affects the clinical treatment and survival of patients.

A phase III randomized study by the Southwest Oncology Group in SCLC patients showed that the combination of CCRT with granulocyte−macrophage colony-stimulating factor (GM-CSF) significantly increased the incidence of toxic events, and the median survival duration was slightly shorter in GM-CSF-treated patients [4]. Therefore, based on this study, the National Comprehensive Cancer Network (NCCN) guidelines, do not recommend prophylactic use of granulocyte colony-stimulating factor (G-CSF) with CCRT; if granulocyte deficiency occurs during radiotherapy, it can be treated routinely with G-CSF. However, the Infectious Diseases Society of America (IDSA) guidelines [5] require that once FN is diagnosed, the treatment must be administered within 2 h because infection may progress very rapidly in patients with granulocyte deficiency. Therefore, the NCCN guidelines removed the non-recommendation for G-CSF prophylaxis during CCRT. In addition, modern precision radiotherapy techniques can significantly reduce toxic effects compared with previous two-dimensional techniques, an d some data from relevant clinical trials show that G-CSF combined with radiotherapy and chemotherapy does not increase chest toxicity and has no effect on survival [6, 7].

Along with modern drug development and the development of newer iterations of G-CSF, the novel agent pegylated recombinant human granulocyte colony-stimulating factor (PEG-rhG-CSF), a long-acting, self-regulating rhG-CSF with reduced plasma clearance and prolonged half-life, warrants further evaluation to determine its value in clinical therapy. Some studies have shown that prophylactic administration of PEG-rhG-CSF reduces the incidence, duration and severity of chemotherapy-associated neutropenia in patients with cervical cancer, breast cancer and other tumors [8–12]. Studies employing PEG-rhG-CSF in SCLC are sparse. To explore whether PEG-rhG-CSF can be applied during CCRT for SCLC, a retrospective, cohort-controlled trial was carried out to evaluate the efficacy and safety in preventing hematological toxicity of PEG-rhG-CSF for SCLC during CCRT.

## Materials and methods

### Study design and inclusion criteria

This was a retrospective cohort study of all patients diagnosed with SCLC and treated with CCRT in the radiotherapy department of Shandong Cancer Hospital from 2013 to 2018. Eighty patients met the inclusion criteria and received PEG-rhG-CSF within 48 h after the end of each cycle of chemotherapy during CCRT. This was defined as prophylactic use, and these patients served as the experimental group. We used a propensity score matching method (the nearest neighbor matching method, caliper value is 0.02) to match 80 patients 1:1 out of 850 patients by various control factors, including age, sex, body mass index (BMI), karnofsky performance status (KPS), history of smoking, history of alcohol consumption, hypertension, diabetes, heart disease, glutathione, glutathione. In addition to meeting the basic inclusion criteria, they did not receive PEG-rhG-CSF treatment within 48 h after the end of chemotherapy during CCRT and only received therapeutic rhG-CSF when bone marrow suppression occurred; therefore, these patients served as the control group.

The inclusion criteria were as follows: (1) patients with pathologically confirmed SCLC who had been treated with 2 cycles of etoposide plus cisplatin (EP) regimen chemotherapy; (2) patients received and completed CCRT and 2 cycles of EP chemotherapy during radiotherapy; (3) patients with an absolute neutrophil count (ANC) < 1 × 10^9^/L after the previous cycle of chemotherapy; (4) patients with a white blood cell (WBC) count > 4 × 10^9^/L and ANC > 2 × 10^9^/L before this CCRT; (5) patients without liver or kidney dysfunction, hematologic disorders or other malignant tumors, aged ≥18 years but ≤70 years, and with KPS of ≥80 points; (6) patients who had not received prior radiotherapy; and (7) patients with complete and traceable imaging data.

### Treatment

Patients in both groups were treated with two cycles of chemotherapy according common regimen in China as follows: etoposide 100 mg/m^2^ ivdrip on days 1 to 5 and cisplatin 40 mg/m^2^ ivdrip on days 1 to 3. The EP regimen was given every 3 weeks as a chemotherapy cycle. Radiotherapy was given at the start of chemotherapy with the same total dose of 60 Gy/30 times, 5 times/week.

The experimental group was injected subcutaneously with 6 mg PEG-rhG-CSF within 48 h after the end of each cycle of chemotherapy during CCRT, while in the control group, PEG-rhG-CSF was not administered prophylactically. If the patients had WBC < 3 × 10^9^/L or ANC < 1 × 10^9^/L during the treatment, 5 μg/kg/d rhG-CSF was injected subcutaneously, and both chemotherapy and radiotherapy were stopped until ANC ≥ 2 × 10^9^/L was achieved.

### Efficacy assessment

(1) Complete blood cell counts, mainly assessing the WBC count, ANC, red blood cell (RBC) count, hemoglobin (HB) level and platelet (PLT) count, were performed every week during CCRT, and the values were compared. (2) The ANCs on days 1, 5, 10, 15 and 20 of the first cycle of concurrent chemotherapy were recorded for comparison between the two groups [13, 14]. (3) Patients with interrupted radiotherapy, radioactive reactions, incidences of FN and adverse reactions were compared between the two groups. (4) Progression-free survival (PFS) was defined as the interval from the first day of CCRT to the first sign of disease progression or death. Overall survival (OS) was determined as the interval from the first day of CCRT to death from any cause. All imaging examinations were re-evaluated to determine progression and record the PFS. OS was analyzed by calling or texting the patients and reviewing patient information. Statistical significance was indicated by *P* < 0.05.

### Statistical analysis

Data analysis was performed using SPSS 24.0 software. For continuous variables, the normality of the two groups was tested by the Shapiro−Wilk (S − W) method. If the data obeyed a normal distribution, they were analyzed by two-independent-sample t-tests and the data were expressed as the mean ± standard deviation. If the data did not conform to a normal distribution, they were analyzed by non-parametric Friedman tests. Classified data were analyzed by the χ^2^ test. *P* < 0.05 indicated that the difference was statistically significant. Categorical variables were expressed as composition ratios.

## Results

### Baseline characteristics of the patients

A total of 160 patients were enrolled in this trial. They were divided into two groups, namely, the PEG-rhG-CSF experimental group and the control group, with 80 participants per group. Table 1 summarizes the baseline characteristics of the patients. The average age of the experimental group was 59.70 years, and that of the control group was 59.33 years. Both groups were predominantly male, and more than half of the patients had a history of smoking. According to the Veterans’ Administration Lung Study Group (VALSG) staging system [15], there were 7 patients with extensive stage disease and 73 patients with limited stage disease in the experimental group. The control group included 8 patients with extensive stage disease and 72 patients with limited stage disease. There was no significant difference between the two groups of patients in various baseline characteristics (*P* > 0.05).

**Table 1:** Patient characteristics at baseline

Characteristic	Experimental group (*N* = 80)	Control group (*N* = 80)	Statistics	P
Age (years)	59.70 ± 8.12	59.33 ± 9.66	t = 0.275	0.784
Sex			χ2 = 0.040	0.841
Male	64	65		
Female	16	15		
Smoking history			χ2 = 0.417	0.519
Smoker	50	46		
Never smoker	30	34		
Stage			χ2 = 0.740	0.786
Extensive stage	7	8		
Limited stage	73	72		

### Blood count differences between the two groups

**Fig. 1:**
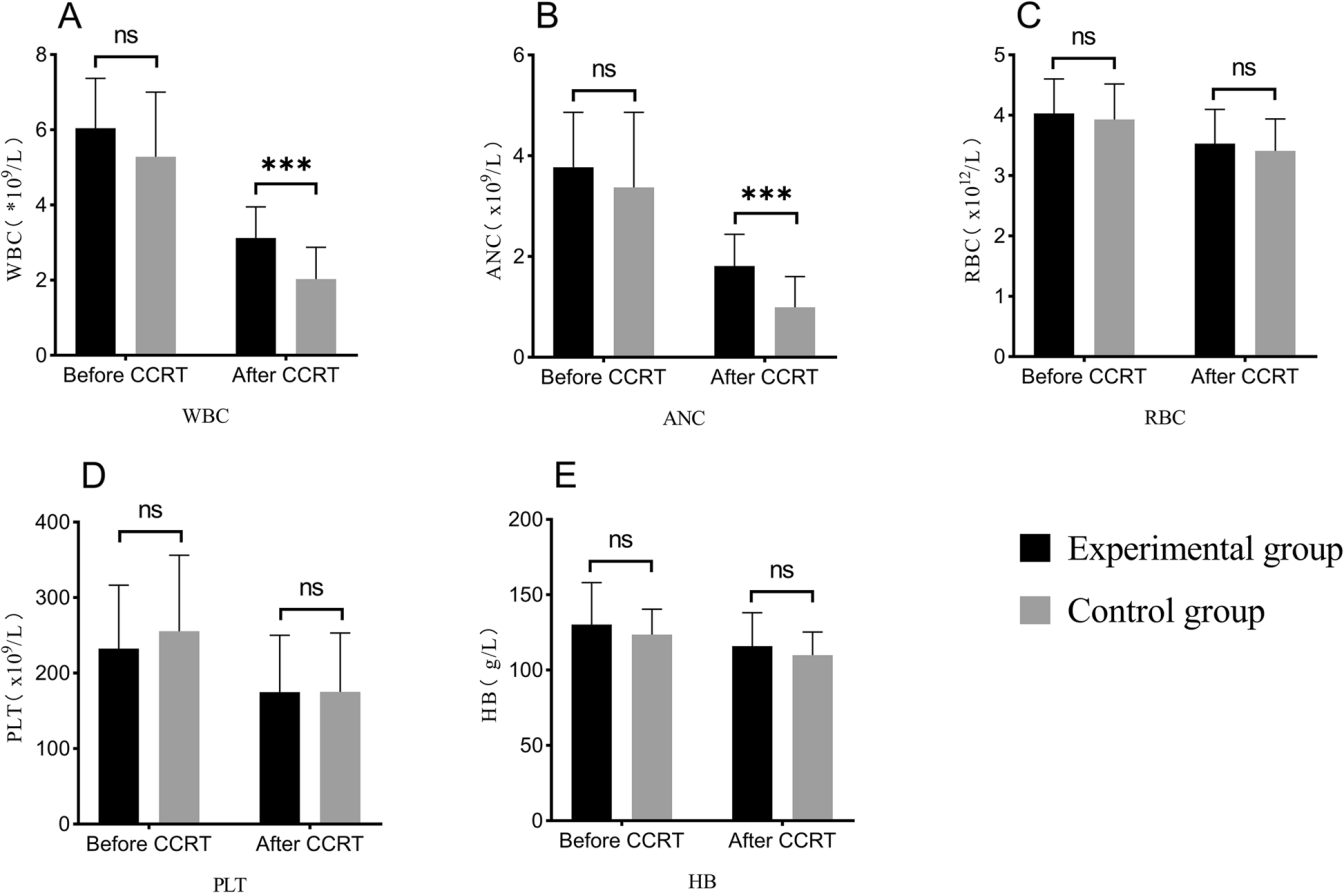
Blood count differences between the two groups. CCRT: concurrent chemoradiotherapy; WBC: white blood cell; ANC: absolute neutrophil count; RBC: red blood cell; PLT: platelet; HB: hemoglobin. * Factors with statistical significance: ns: no significance; *: *p* < 0.05; **: *p* < 0.01; ***: *p* < 0.001

There were no significant differences between the two groups in WBC count, ANC, RBC count, PLT count or HB level before CCRT (*P* > 0.05). However, after CCRT, the counts decreased to varying degrees, and the WBC count and ANC of the experimental group were slightly higher than those of the control group; the difference was significant (*P* < 0.05). The RBC count, PLT count and HB level showed no significant changes after CCRT, as shown in Fig. 1A-E (Supplementary Table 1).

### Changes in ANC

**Fig. 2:**
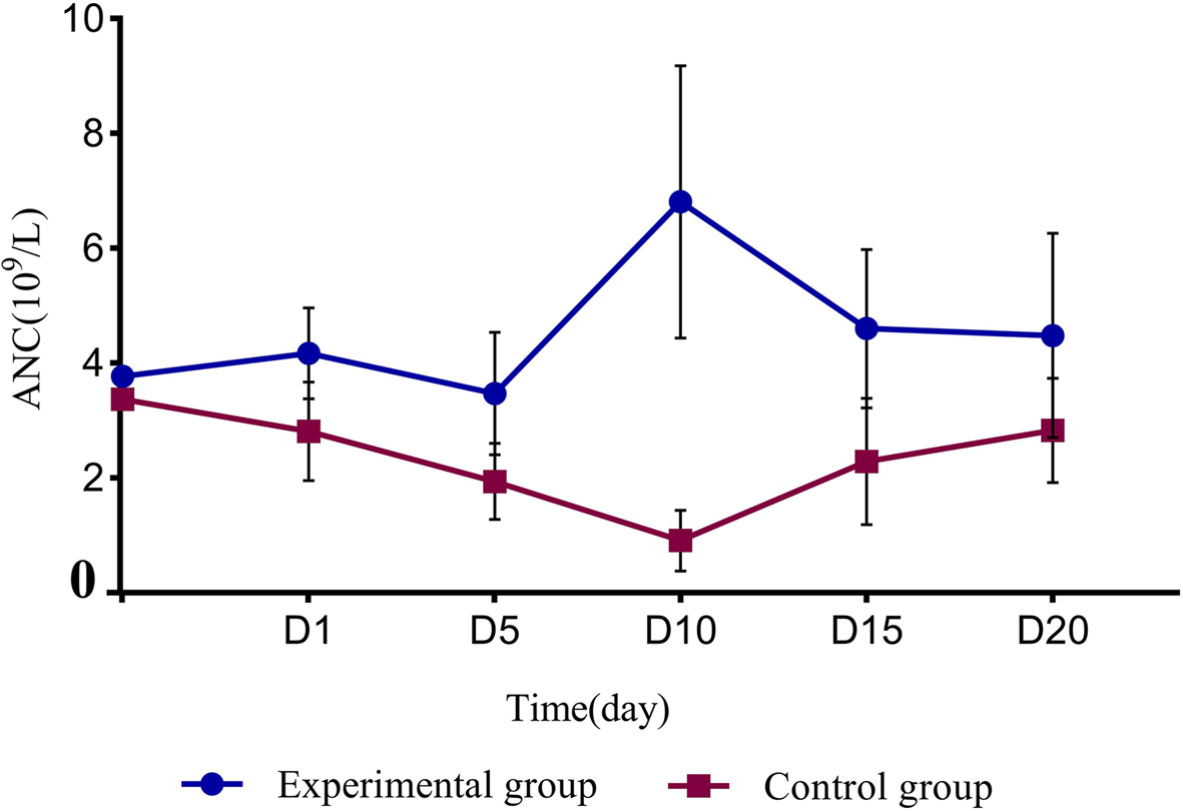
ANC dynamics in the two groups

Figure 2 show the changes in ANC on days 1, 5, 10, 15, and 20 (D1, D5, D10, D15, and D20, respectively) in both groups of patients. The ANC was 4.17 ± 0.79 (× 10^9^/L) in the experimental group and 2.81 ± 0.86 (× 10^9^/L) in the control group on D1. It reached a peak of 6.81 ± 2.37 (× 10^9^/L) on D10 after the administration of PEG-rhG-CSF in the experimental group, while it decreased significantly in the control group, reaching a minimum of 0.91 ± 0.53 (× 10^9^/L); the difference was statistically significant (*P* < 0.05; Supplementary Table 2). During CCRT, although rhG-CSF was given when grade III or higher neutropenia occurred in the control group, the ANC was still lower than that in the experimental group. During the treatment, a total of 15 people in the experimental group had delayed treatment because of granulocytopenia, compared to 29 people in the control group. The difference was significant (*P* = 0.013).

### Hematological toxicity

**Fig. 3:**
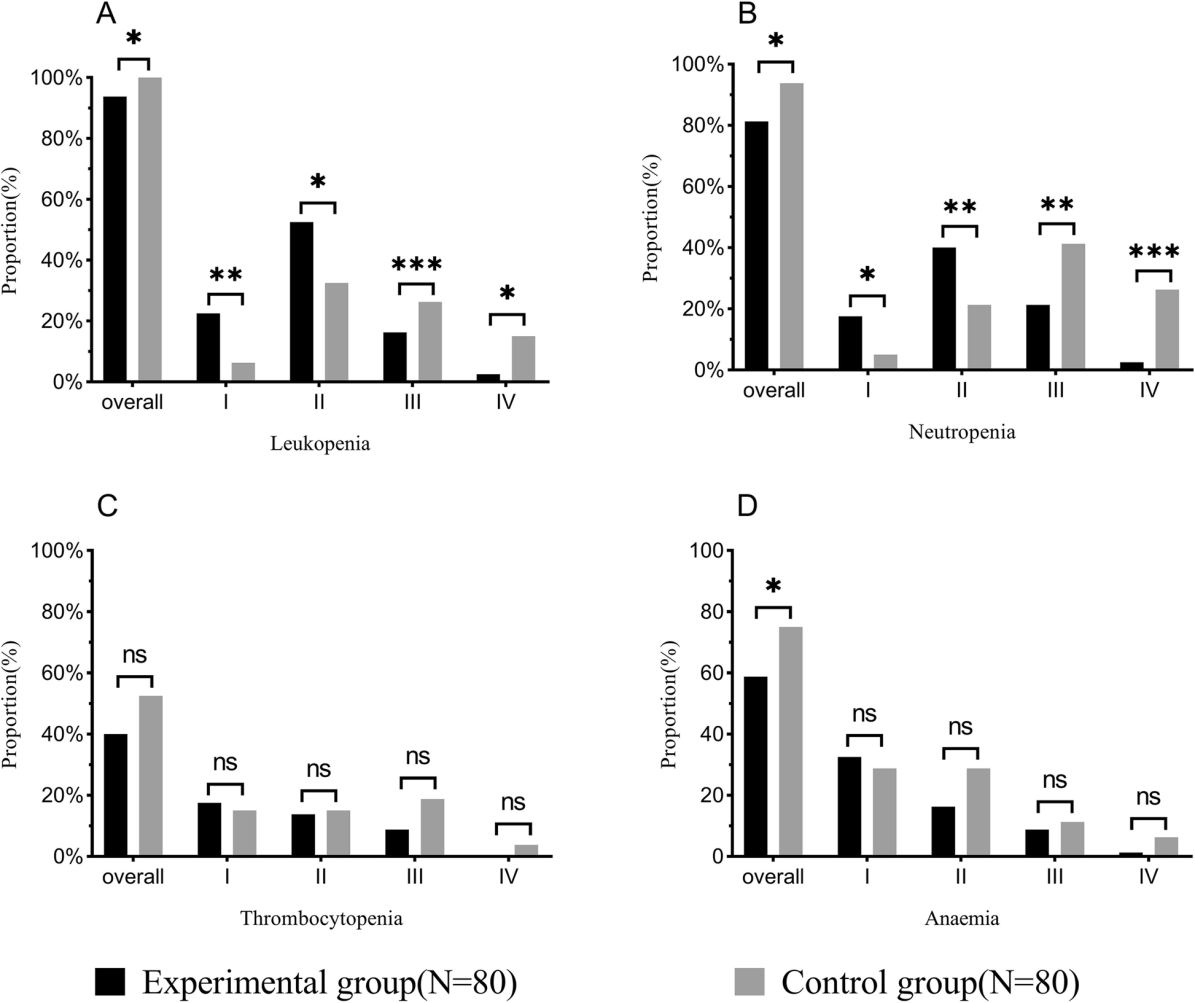
Differences in myelosuppression. * Factors with statistical significance: ns: no significance; *: *p* < 0.05; **: *p* < 0.01; ***: *p* < 0.001

According to the World Health Organization’s criteria for acute and subacute toxic reactions, post-chemotherapy myelosuppression is classified as grade 0-IV [16]. The hematological toxicity data are shown in Fig. 3A-D (Supplementary Table 3). The total incidences of leukopenia (93.75% vs. 100.0%) and neutropenia (81.25% vs. 93.75%) in the experimental group were lower than those in the control group (*P* < 0.05), and the incidences of grade III-IV leukopenia (18.75% vs. 61.25%) and neutropenia (23.75% vs. 67.5%) in the experimental group were also significantly lower than those in the control group (*P* < 0.05). However, there were no significant differences in the incidence of grade III-IV thrombocytopenia (8.75% vs. 22.5%) or anemia (10.0% vs. 17.5%) between the two groups (*P* > 0.05).

### Adverse reactions

**Fig. 4:**
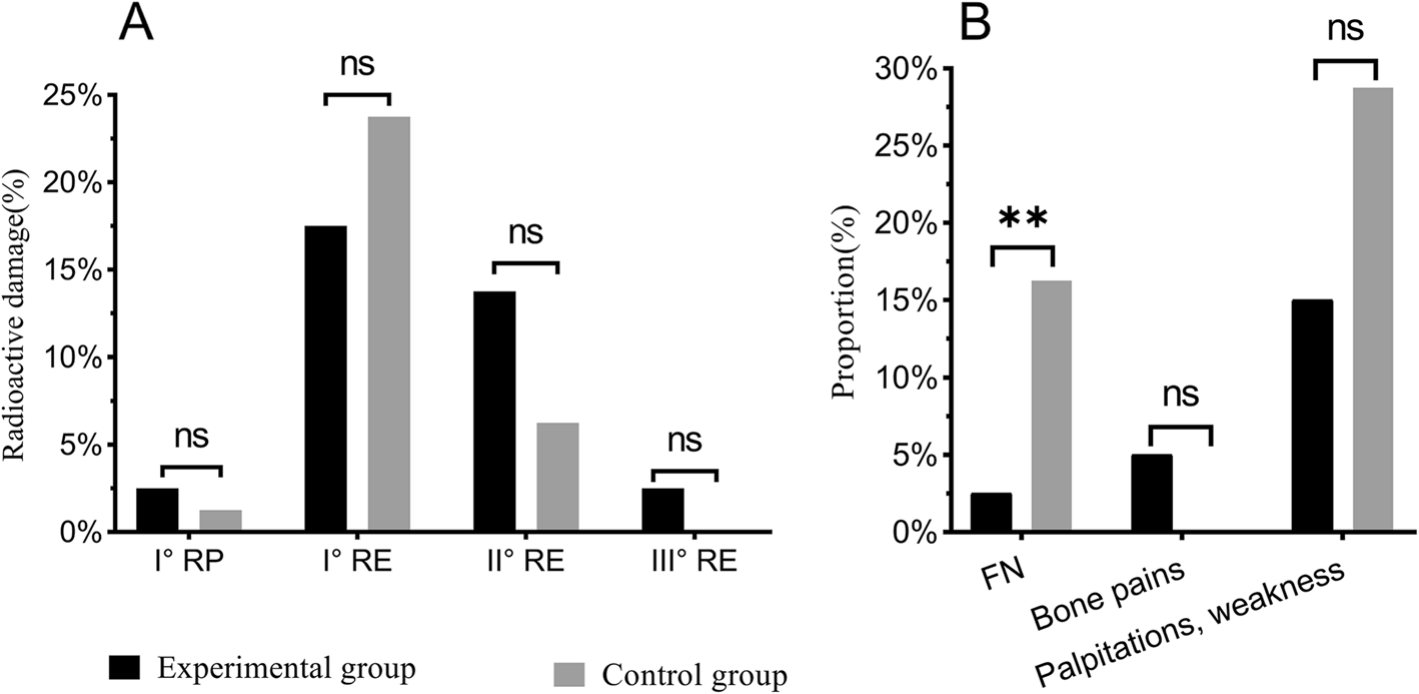
Radiological reactions, incidence of FN and other adverse reactions. RP: Radiation pneumonia, RE: Radiation esophagitis, FN: Febrile neutropenia. * Factors with statistical significance: ns: no significance; *: *p* < 0.05; **: *p* < 0.01; ***: *p* < 0.001

The main side effects of treatment in all patients were radiation pneumonia and esophagitis. Based on the Radiation Therapy Oncology Group (RTOG) grading scale for acute radiation injury [17], 2 patients in the experimental group and 1 patient in the control group developed 1st-degree radiation pneumonia. The number of patients with 1st-, 2nd- and 3rd-degree esophagitis was 14, 11 and 2 in the experimental group and 19, 5 and 0 in the control group, respectively. None of the differences were significant (*P* > 0.05), as shown in Fig. 4A. FN occurred in 2 patients (2.5%) in the experimental group and occurred in 13 patients (16.25%) in the control group, and the incidence was significantly lower in the experimental group than in the control group (*P* = 0.003). Other adverse reactions of patients in both groups were mainly bone pain, palpitation and weakness, and the differences were not statistically significant (*P* > 0.05). Patients with bone pain had relatively mild symptoms that dissipated without intervention, and other adverse reactions were relieved after discontinuation of the drug and administration of symptomatic treatment, as shown in Fig. 4B (Supplementary Table 4).

### Survival

**Fig. 5:**
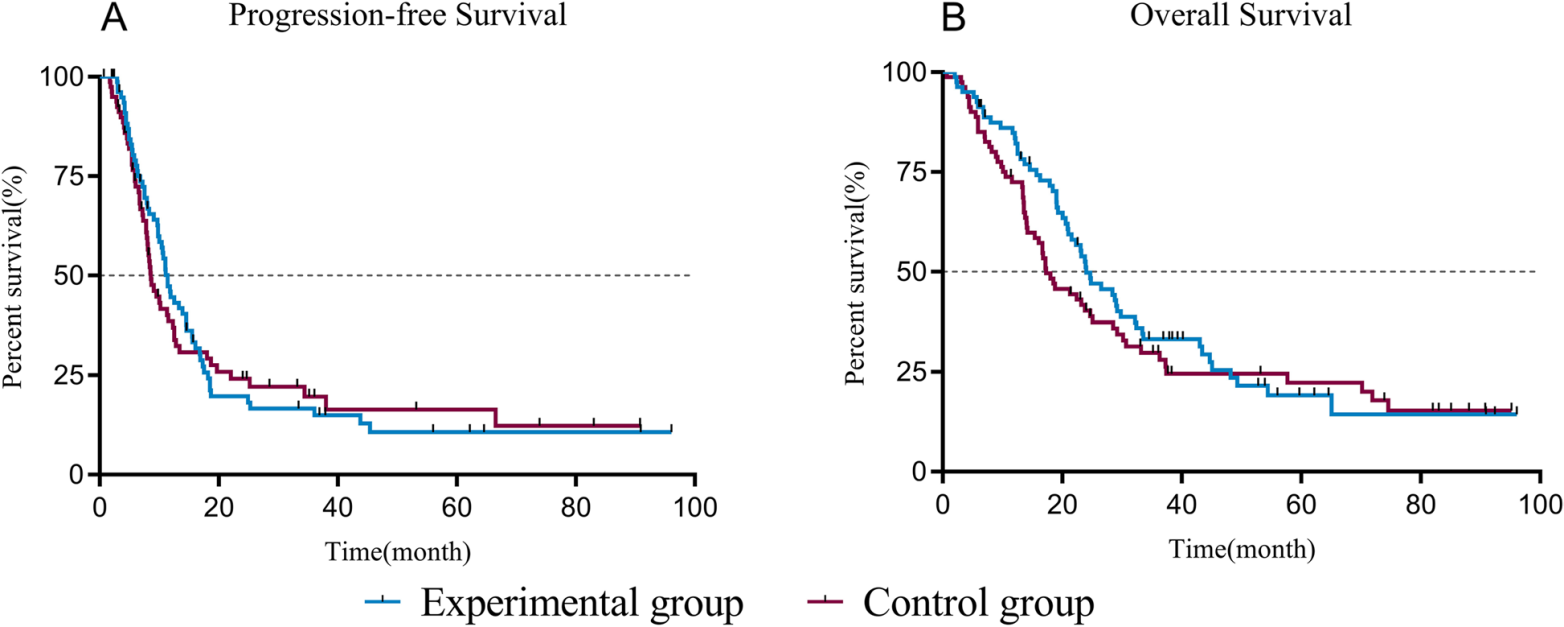
Kaplan-Meier curves of PFS (**A**) and OS (**B**) of the two groups

A total of 13 people were lost to follow-up, namely, 9 in the experimental group and 4 in the control group. The follow-up period was 0.7–96.1 months as of May 1, 2021, with a median follow-up period of 18.6 months. Prophylactic application of PEG-rhG-CSF during CCRT had no significant effects on PFS (11.4 months vs. 8.7 months; *P* = 0.756) or OS (23.9 months vs. 17.3 months; *P* = 0.325). The differences were not statistically significant, as shown in Fig. 5A, B.

## Discussion

The most common side effect during CCRT is blood cell toxicity. In severe cases, it can induce FN, and the immune decline triggers infections and can even be life-threatening [18, 19]. At present, rhG-CSF and PEG-rhG-CSF are routinely used in antitumor adjuvant therapy, and PEG-rhG-CSF has shown more strengths. A large number of domestic and international studies have confirmed that PEG-rhG-CSF has similar efficacy to rhG-CSF but has superior safety in the prophylactic treatment of chemotherapy-induced non-myeloid-derived neutropenia [20–24]. RhG-CSF requires daily administration because of its short half-life [25–27]. However, PEG-rhG-CSF is a long-acting, self-regulating rhG-CSF that has reduced plasma clearance and prolonged half-life compared to rhG-CSF, with only one dose required per chemotherapy cycle [26, 28, 29]. Therefore, it is more favorable for clinical treatment.

Research has shown that concurrent use of chemotherapy and administration of hematological growth factors may enhance hematological toxicity [30]. The initial evidence came from a multicenter prospective trial by the Southwest Oncology Group [4]. The results show that patients administered GM-CSF had higher WBC counts and ANCs, but there was no significant difference in the incidence of grade IV leukopenia or neutropenia. However, there was a statistically significant increase in the incidences of thrombocytopenia and anemia in the GM-CSF arm. The rates of infection and toxicity-related death were also higher in GM-CSF patients.

Early studies focused on GM-CSF, which does not act specifically on granulocyte progenitors, and it is no longer a routine treatment used to raise leukocyte counts. Therefore, it is necessary to re-evaluate the safety of granulocyte-stimulating factors. Some studies have shown that the use of granulocyte-stimulating factors is justified [31]. In addition, a randomized controlled trial and meta-analysis indicated that the incidences of neutrophil deficiency and FN were significantly improved with the use of long-acting G-CSF [32]. Goodman Lindsey Martin et al. analyzed the prophylactic use of PEG-rhG-CSF in 180 patients with NSCLC receiving new chemotherapy regimens and showed a significant reduction in the FN risk [33]. In the past, G-CSF was given to patients deemed to be at high risk of developing hematological toxicity. However, the results of the available studies contradict previous perceptions. Patients treated with G-CSF have significantly lower hematological toxicity and may have better treatment efficacy. The results of our trial further confirm this.

A phase II clinical trial of prophylactic application of G-CSF in 38 patients with limited-stage SCLC treated with CCRT did not show an increased risk of acute or advanced pulmonary toxicity or grade III-IV acute esophagitis, nor did it result in treatment-related mortality in patients. There was no significant difference in the incidence of neutropenia, but the incidence of grade III/IV adverse events was slightly lower than that in the non-preventive use group. However, prophylactic application of G-CSF increased the risk of thrombocytopenia [7]. This result is similar to that of the trial by the Southwest Oncology Group. Our study suggests that the PLT counts is decreased with PEG-rhG-CSF administration, and the decrease after therapy was still lower than that in patients not treated with PEG-rhG-CSF. The grade III-IV thrombocytopenia incidence was lower than in patients who did not use PEG-rhG-CSF. Although there was no difference in the PLT count, there were also no bleeding events due to thrombocytopenia during the treatment. Therefore, the reduction in the PLT count may be a manifestation of myelosuppression and not closely related to PEG-rhG-CSF administration. CONVERT is a phase III randomized controlled trial involving 547 patients with SCLC treated with CCRT [6]. Of the patients, 33% received at least one cycle of prophylactic G-CSF, and 41% received therapeutic G-CSF. The application of G-CSF during CCRT did not increase the risk of acute esophagitis or pulmonary toxicity and facilitated treatment completion. In contrast, although all patients had imaging examinations every 2–3 months, few patients developed radiation pneumonia and no severe radiation esophagitis (predominantly grade I and II esophagitis) in our study. This may be due to the low frequency of chest X-ray or Chest CT scan, and some patients are lost to follow-up. This makes it difficult to determine whether radioactive pneumonia data occurred. This study is after all a retrospective clinical study with a small sample size and certain limitations, but these findings can show that the safety and efficacy of PEG-rhG-CSF in hematology is reliable. Therefore, it is feasible to prophylactically administer granulocyte-stimulating factor. Previous studies by the Southwest Oncology Group have shown that the median survival duration is slightly shorter in GM-CSF-treated patients than in patients treated without GM-CSF. However, G.H. Lyman et al. conducted a systematic review and meta-analysis of the effect of G-CSF on the chemotherapy dose and survival of oncology patients [34]. Patients treated with G-CSF had reduced all-cause mortality. Atsuto Mouri performed a retrospective study of 33 patients with non-small-cell lung cancer (NSCLC) who had previously developed FN, and 29 patients received PEG-x’rhG-CSF prophylactically at the start of the next cycle of chemotherapy [20]. The median PFS and OS times for patients treated with and without PEG-rhG-CSF prophylactically were 177 and 163 days (PFS; *P* = 0.20), 628 days and 274 days (OS; *P* = 0.13), respectively, which are similar to the results of our study. These results further illustrate the reliability of PEG-rhG-CSF. Prophylactic use of PEG-rhG-CSF may reduce the probability of radiotherapy-related adverse events and make oncology treatment safe and effective. More importantly, it had no significant impact on survival time.

Our study focused on hematological toxicity with PEG-rhG-CSF administration during CCRT. The results showed that prophylactic application of PEG-rhG-CSF significantly reduced the incidences of leukopenia and neutropenia, especially the incidences of grade III-IV leukopenia and neutropenia. More importantly, there were no significant differences in the incidences of grade III-IV thrombocytopenia and anemia between the groups, and there were no cases of blood transfusion, death or other serious adverse events. Furthermore, on the 10th day after chemotherapy, the experimental group achieved the peak ANC and thereafter maintained normal neutrophil levels, while the control group showed varying degrees of reduction despite regular administration of rhG-CSF. Importantly, the number of patients with radiotherapy interruptions was significantly lower in the experimental group than in the control group. In addition, the incidence of FN was also significantly decreased, and the incidences of all other adverse reactions were within the control ranges. There was also no significant acute esophagitis or pulmonary toxicity. Furthermore, no toxicity-related deaths were observed, and the results showed no effects on PFS or OS. These findings show that PEG-rhG-CSF has good reliability and compliance.

## Conclusion

The prophylactic administration of PEG-rhG-CSF can significantly reduce the incidences of leukopenia and neutropenia, especially the incidence of grade 3 or higher hemocytopenia events, and can significantly reduce the incidence of FN and the frequency of treatment interruptions. More importantly, prophylactic administration of PEG-rhG-CSF did not lead to a significant effect on survival time. This result indicates that PEG-rhG-CSF has good efficacy and safety in preventing hematological toxicity in SCLC patients during CCRT. The clinical analysis of this study remains somewhat biased due to case selection, small sample size, and the limitations of being a retrospective cohort study; therefore, further validation with a larger sample or related prospective studies are needed. However, the results of this article are also informative for clinical guidance.

## Supplementary Information

**Additional file 1: S1 Table.** Blood counts differences. These data did not obey a normal distribution according to the Shapiro−Wilk test (*P* < 0.05), so nonparametric tests on two independent samples were used for comparison. **S2 Table.** Changes in ANC (× 109/L). The data, which did not obey the normal distribution according to the Shapiro−Wilk test (*P*<0.05), so a nonparametric (Friedman) test was used. **S3 Table.** Hematological toxicity incidence in the two groups (% patients). **S4 Table.** Incidence of FN and other adverse reactions in the two groups. FN: febrile neutropenia

## Data Availability

The datasets generated during and analyzed during the current study are not publicly available due to privacy or ethical restrictions but are available from the corresponding author on reasonable request.

## Abbreviations

PEG-rhG-CSF: Pegylated recombinant human granulocyte colony-stimulating factor
CCRT: Concurrent chemoradiotherapy
SCLC: Small-cell lung cancer
FN: Febrile neutropenia
PFS: Progression-free survival
OS: Overall survival
GM-CSF: Granulocyte−macrophage colony-stimulating factor
NCCN: National comprehensive cancer network
G-CSF: Granulocyte colony-stimulating factor
IDSA: Infectious diseases society of America
ANC: Absolute neutrophil count
KPS: Karnofsky performance status
WBC: White blood cell
RBC: Red blood cell
PLT: Platelet
HB: Hemoglobin
RP: Radiation pneumonia
RE: Radiation esophagitis
BMI: body mass index
