# Supplementary material for: Safety and efficacy of pegylated recombinant human granulocyte colony-stimulating factor during concurrent chemoradiotherapy for small-cell lung cancer: a retrospective, cohort-controlled trial
Source: BMC Cancer. 2022 May 13;22:542. doi: 10.1186/s12885-022-09644-8 (PMC9107159; doi:10.1186/s12885-022-09644-8)
Supplement: Supplementary file 1 — Additional file 1: S1 Table. Blood counts differences. These data did not obey a normal distribution according to the Shapiro−Wilk test (P < 0.05), so nonparametric tests on two independent samples were used for comparison. S2 Table. Changes in ANC (× 109/L). The data, which did not obey the normal distribution according to the Shapiro−Wilk test (P<0.05), so a nonparametric (Friedman) test was used. S3 Table. Hematological toxicity incidence in the two groups (% patients). S4 Table. Incidence of FN and other adverse reactions in the two groups. FN: febrile neutropenia [file 12885_2022_9644_MOESM1_ESM.doc]

Table 1 Blood counts differences.

|  | Before CCRT (N=80) | After CCRT (N=80) | Z | P |
| --- | --- | --- | --- | --- |
| Experimental group |  |  |  |  |
| WBC count (*10^9^/L) | 6.04±1.33 | 3.12±0.83 | -10.421 | <0.001 |
| ANC (x10^9^/L) | 3.77±1.09 | 1.81±0.63 | -9.757 | <0.001 |
| RBC count (x10^12^/L) | 4.03±0.57 | 3.53±0.57 | -5.326 | <0.001 |
| PLT count (x10^9^/L) | 232.49±83.91 | 174.74±75.30 | -4.491 | <0.001 |
| HB (g/L) | 130.18±27.93 | 115.93±22.08 | -4.404 | <0.001 |
| Control group |  |  |  |  |
| WBC count (x10^9^/L) | 5.28±1.72 | 2.03±0.85 | -10.624 | <0.001 |
| ANC (x10^9^/L) | 3.37±1.49 | 0.99±0.61 | -10.301 | <0.001 |
| RBC count (x10^12^/L) | 3.93±0.59 | 3.41±0.53 | -5.152 | <0.001 |
| PLT count (x10^9^/L) | 255.46±100.59 | 175.16±78.04 | -5.317 | <0.001 |
| HB (g/L) | 123.50±16.92 | 109.89±15.40 | -4.724 | <0.001 |
| Z |  |  |  |  |
| WBC count | -1.903 | -6.901 |  |  |
| ANC | -1.908 | -7.356 |  |  |
| RBC count | -1.085 | -1.604 |  |  |
| PLT count | -1.111 | -0.092 |  |  |
| HB | -1.162 | -1.751 |  |  |
| P |  |  |  |  |
| WBC count | 0.057 | <0.001 |  |  |
| ANC | 0.056 | <0.001 |  |  |
| RBC count | 0.278 | 0.109 |  |  |
| PLT count | 0.267 | 0.927 |  |  |
| HB | 0.245 | 0.080 |  |  |

These data did not obey a normal distribution according to the Shapiro−Wilk test (P<0.05), so nonparametric tests on two independent samples were used for comparison.

CCRT: concurrent chemoradiotherapy; WBC: white blood cell; ANC: absolute neutrophil count; RBC: red blood cell; PLT: platelet; HB: hemoglobin.

Table 2 Changes in ANC (x10^9^/L)

| Group | Day of observation | | | | |
| --- | --- | --- | --- | --- | --- |
|  | D1 | D5 | D10 | D15 | D20 |
| Experimental group | 4.17±0.79 | 3.47±1.07 | 6.81±2.37 | 4.60±1.38 | 4.48±1.78 |
| Control group | 2.81±0.86 | 1.94±0.66 | 0.91±0.53 | 2.29±1.10 | 2.83±0.91 |
| Z | -8.320 | -8.904 | -10.808 | -9.443 | -7.592 |
| P | <0.001 | <0.001 | <0.001 | <0.001 | <0.001 |

The data, which did not obey the normal distribution according to the Shapiro−Wilk test (P＜0.05), so a nonparametric (Friedman) test was used.

Table 3 Hematological toxicity incidence in the two groups (% patients)

|  | Leukopenia | Neutropenia | Thrombocytopenia | Anemia |
| --- | --- | --- | --- | --- |
| Experimental group  (N=80) |  |  |  |  |
| Overall (%) | 75  (93.75) | 65  (81.25) | 32  (40.0) | 47  (58.75) |
| I (%) | 18  (22.5) | 14  (17.5) | 14  (17.5) | 26  (32.5) |
| II (%) | 42  (52.5) | 32  (40.0) | 11  (13.75) | 13  (16.25) |
| III (%) | 13  (16.25) | 17  (21.25) | 7  (8.75) | 7  (8.75) |
| IV (%) | 2  (2.5) | 2  (2.5) | - | 1  (1.25) |
| Control group  (N=80) |  |  |  |  |
| Overall (%) | 80  (100) | 75  (93.75) | 42  (52.5) | 60  (75.0) |
| I (%) | 5  (6.25) | 4  (5.0) | 12  (15.0) | 23  (28.75) |
| II (%) | 26  (32.5) | 17  (21.25) | 12  (15.0) | 23  (28.75) |
| III (%) | 37  (46.25) | 33  (41.25) | 15  (18.75) | 9  (11.25) |
| IV (%) | 12  (15.0) | 21  (26.25) | 3  (3.75) | 5  (6.25) |
| P | P<0.05 | P<0.05 | P>0.05 | P>0.05 |

Table 4 Incidence of FN and other adverse reactions in the two groups

| Adverse reaction | Experimental group  (N=80) | Control group  (N=80) | χ^2^ | P |
| --- | --- | --- | --- | --- |
| FN | 2 (2.5%) | 13 (16.25%) | 8.901 | 0.003 |
| Bone pain | 4 (5.0%) | 0 (0.0%) | 4.103 | 0.120 |
| Palpitations or weakness | 12 (15.0%) | 23 (28.75%) | 4.425 | 0.055 |

FN: febrile neutropenia
